# Cognitive telerehabilitation: an expert consensus paper on current evidence and future perspective

**DOI:** 10.3389/fneur.2024.1338873

**Published:** 2024-02-15

**Authors:** Maria Grazia Maggio, Francesca Baglio, Francesco Arcuri, Francesca Borgnis, Marianna Contrada, Marcos Daniel Maldonado Diaz, Carl Froilan Leochico, Nicolas Jimenez Neira, Stefania Laratta, Boris Suchan, Paolo Tonin, Rocco Salvatore Calabrò

**Affiliations:** ^1^IRCCS Centro Neurolesi “Bonino Pulejo”, Messina, Italy; ^2^IRCCS Fondazione Don Carlo Gnocchi Onlus, Milan, Italy; ^3^S. Anna Institute and Research in Advanced Neurorehabilitation, Crotone, Italy; ^4^Clínica Alemana, Universidad del Desarrollo, Santiago, Chile; ^5^University of the Philippines Manila, Manila, Philippines; ^6^St. Luke’s Medical Center, Quezon City, Philippines; ^7^Fundación Polibea, Madrid, Spain; ^8^Department of Neuropsychology, Institute of Cognitive Neuroscience, Faculty of Psychology, Ruhr-Universität Bochum, Bochum, Germany

**Keywords:** cognitive rehabilitation, neurological diseases, neurorehabilitation, remote rehabilitation, telerehabilitation

## Abstract

The progressive improvement of the living conditions and medical care of the population in industrialized countries has led to improvement in healthcare interventions, including rehabilitation. From this perspective, Telerehabilitation (TR) plays an important role. TR consists of the application of telemedicine to rehabilitation to offer remote rehabilitation services to the population unable to reach healthcare. TR integrates therapy-recovery-assistance, with continuity of treatments, aimed at neurological and psychological recovery, involving the patient in a family environment, with an active role also of the caregivers. This leads to reduced healthcare costs and improves the continuity of specialist care, as well as showing efficacy for the treatment of cognitive disorders, and leading to advantages for patients and their families, such as avoiding travel, reducing associated costs, improving the frequency, continuity, and comfort of performing the rehabilitation in its own spaces, times and arrangements. The aim of this consensus paper is to investigate the current evidence on the use and effectiveness of TR in the cognitive field, trying to also suggest some recommendations and future perspectives. To the best of our knowledge, this is the first consensus paper among multiple expert researchers that comprehensively examines TR in different neurological diseases. Our results supported the efficacy and feasibility of TR with good adherence and no adverse events among patients. Our consensus summarizes the current evidence for the application of cognitive TR in neurological populations, highlighting the potential of this tool, but also the limitations that need to be explored further.

## 1 Introduction

The progressive improvement of the living conditions and medical care of the population in industrialized countries has led to an improvement in healthcare interventions, including rehabilitation. In particular, some studies have shown that neurological patients need continuous rehabilitation treatments that healthcare facilities struggle to provide, due to organizational difficulties and excessive costs. This situation, especially following the COVID-19 pandemic, has stimulated new models for the provision of rehabilitation services, such as remote rehabilitation, favoring non-hospital services with continuity of care.

Telerehabilitation (TR) consists of the application of telemedicine to rehabilitation to offer remote rehabilitation services to the population unable to reach healthcare facilities. Healthcare services are provided through information and communication technologies, in situations where the healthcare worker and the patient are not in the same physical location (1). This occurs through the secure transmission of data and multimedia information, such as texts, sounds, images, and other files, in order to provide a variety of services. TR reduces healthcare costs and expenses, with a shortening of hospitalization times (2). It eliminates the time and stress of the transfer of patients to hospital services, especially for those who are far away from the main facilities, guaranteeing home access to physical and cognitive training. Moreover, it promotes patient-professional interaction in real-time and adapts the rehabilitation times to the patient’s abilities, allowing for good patient compliance. TR integrates therapy-recovery-assistance, with continuity of treatments, aimed at neurological and psychological recovery, involving the patient in a family environment, with an active role also of the caregivers. Various studies have shown that TR can positively affect neurological patients, with results comparable to standard care (3–5). In particular, TR treatments can activate cortical regions similar to those activated by conventional treatment. The data of the literature underline that this innovative means can have promising results in the neurological rehabilitation of the elderly, and adults with different cognitive disorders (1–5).

Cognitive impairment is defined as disturbances in thinking, learning, memory, judgment, and decision-making. Signs of cognitive decline include memory loss and difficulty concentrating, completing tasks, understanding, remembering, following directions, and problem-solving. Other common signs are changes in mood or behavior, loss of motivation, and disorientation (6). Cognitive impairments can be found both in patients with brain injuries (traumatic or vascular) and in patients with neurodegenerative diseases, which have a progressive course that finally culminates in global cognitive impairment and impaired functional independence (3). Treatments such as home-based TR have appeared as an alternative to institutionalized rehabilitation (7). This leads to reduced healthcare costs and improves the continuity of specialist care, as well as showing efficacy for the treatment of cognitive disorders, and leading to advantages for patients and their families, such as avoiding travel, reducing associated costs, improving the frequency, continuity, and comfort of performing the rehabilitation in its own spaces, times and arrangements (3).

The literature reports that TR appears to be a promising alternative. Various studies could support the effectiveness of TR and its application for the treatment of cognitive problems in neurological pathologies (3). In particular, some studies have shown improvements in executive function supported by consistent evidence of increases in attention spans, memory capacities, and quality of life with a more rapid return to independence (1–4). Despite this, there is insufficient evidence to conclude the superiority of TR training in the global cognitive domain, language functions, and learning and memory abilities (3). Evidence related to the effect of telerehabilitation programs performed in combination with virtual reality (VR) training on different cognition domains and their tolerability in neurological patients indicated a key role of these tools in the prevention and treatment of cognitive impairment. Previous studies have shown that dual tasks (cognitive training and exercise) could have positive effects on cognitive function among older adults (8). Previous studies have shown that dual tasks (cognitive training and exercise) could have positive effects on cognitive function among older adults (1, 8). Therefore, cognitive telerehabilitation is an emerging modality for the delivery of cognitive, motor, or linguistic treatments, in which the need to ensure continuity of care is emphasized. Especially in combination with VR, TR has the potential to ease access to services and provide continuity of treatment, without reducing its intensity and frequency after hospital discharge and thus ease patients with their disturbing condition and contribute to treatment timely and effectively.

The aim of this consensus paper is to investigate the current evidence on the use and effectiveness of TR in the cognitive field, trying to also suggest some recommendations and future perspectives.

## 2 Addressing challenges in cognitive tele-rehabilitation: a comprehensive exploration

In navigating the landscape of cognitive tele-rehabilitation, it is essential to begin by candidly delineating the multifaceted limitations that characterize this treatment modality. Indeed, cognitive telerehabilitation, although promising, faces several challenges that must be carefully considered to ensure its success and effectiveness. Limited technological accessibility is a primary obstacle, as not all patients may have the necessary devices or access to a reliable internet connection. This technological gap could be particularly pronounced in certain demographic groups or socio-economic contexts. Additionally, different technical skills among patients create a potential barrier to the utilization of virtual platforms.

Environmental disruptions in the home context, such as noise or distractions, can negatively impact the patient’s concentration during virtual rehabilitation sessions. The lack of direct physical interaction with healthcare providers represents a limitation in the detailed observation of the patient’s conditions and the customization of treatment.

Therefore, the active involvement of caregivers is crucial to ensuring a successful experience in cognitive telerehabilitation. In the virtual context, the caregiver plays a critical support role, helping mitigate technological challenges and ensuring that the patient follows the treatment adequately. Collaboration among patients, caregivers, and healthcare providers becomes essential to overcome barriers and maximize the benefits of cognitive telerehabilitation.

Beyond practical issues, cultural variations can influence the perception and adoption of telerehabilitation, requiring a sensitive approach to cultural differences. Moreover, concerns related to privacy and data security could undermine patient trust in the use of virtual platforms.

Another significant limitation is the need to carefully assess patients to determine their eligibility for inclusion in cognitive telerehabilitation programs. Indeed, a rigorous process of leveling the patient to see whether or not they have the cognitive capacity to access/benefit from a virtual platform is fundamental. This evaluation process, if not executed accurately, could compromise the overall effectiveness of treatment, emphasizing the importance of a precise assessment to ensure optimal results and safe patient participation.

In conclusion, to fully unlock the potential of cognitive telerehabilitation, it is imperative to meticulously address the identified limitations, formulating targeted strategies and solutions that guarantee an inclusive, effective, and safe experience for all patients. It is important to note that these complexities may pose challenges in establishing definitive recommendations, requiring ongoing research and adaptation to navigate the evolving landscape of cognitive telerehabilitation effectively.

## 3 Methods

### 3.1 Approach to the Delphi process

We employed the Delphi method to achieve consensus among clinicians regarding the current evidence and future perspectives of cognitive telerehabilitation. This method is increasingly utilized to develop clinical practice guidelines and define core outcome sets for clinical trials (9).

### 3.2 Consensus building

The inaugural meeting of the research group, conducted via a web conference on April 26, 2021, with 12 participants, aimed to define the purpose of a narrative review focusing on the state of cognitive telerehabilitation across various pathologies. Authors were selected based on their expertise in the field, evaluated through parameters such as the h-index and publications related to consensus building. During this meeting, it was decided to form working groups, each composed of three individuals, to address specific pathologies based on their respective areas of expertise. The subgroups were as follows:

- Devices for TR: FA, MC, and PT.
- Cognitive TR and stroke: MD, MM, and BS.
- Telerehabilitation and cognitive-behavioral problems in TBI: MM, MD, and CL.
- MCI, dementia and TR: FBa, FBo, and NN.
- Cognitive TR and multiple sclerosis: FBa, FBo, and MM.
- Cognitive TR in PD: MM, RC, and PT.
- Introduction: MM, RC, and PT.
- Discussion: MM, FBa, FA, FBo, MC, MD, CL, NN, SL, BS, PT, and RC.

Another web conference meeting took place on July 13, 2021, during which each subgroup determined the articles to be included in the review, and necessary resolutions were discussed. All authors assessed the articles for inclusion based on relevance, citation rates, and alignment with the review objectives.

A third meeting occurred on May 26, 2022, in the form of a web conference (attended by 12 members) to consolidate consensus on the content written by each group and to delve deeper into the discussion.

Finally, a concluding meeting was held on March 15, 2023, to approve the final version of the manuscript. The Likert scale, implemented during the consensus evaluation, served as a numerical representation of participants’ agreement on various aspects, including information consistency, drafting method, and adequacy of inserted sentences, especially focusing on the inclusion of articles in the review. Participants, maintaining anonymity, in line with the Delphi criteria, rated their agreement on a 10-point Likert scale, with 10 indicating the highest agreement. The consensus threshold was set at more than 85%, ensuring robust agreement among the research group. Coordination and data collection during the meetings were led by MM, PT, and RC. The voting results were reiterated, necessary changes were implemented, and final approval was obtained from all participants.

The main results are summarized in Table 1.

**Table 1:** Shows the main studies concerning cognitive telerehabilitation.

Type	Study	Study’s design	Patients	Device and training	Major findings
Stroke and TR	Torrisi et al. (10)	Exploratory study	40 patients affected by stroke	Tablet training at home 2 times a week	The authors observed that VR improved global cognitive functioning and semantic and phonetic fluency
	Lawson et al. (11)	Exploratory study	46 stroke patients	Zoom video conferencing and face-to-face training	The authors observed that TR was a feasible and effective method, as it could increase compensatory distance memory capacity to a greater extent than face-to-face rehabilitation
	Faria et al. (12)	Exploratory study	36 stroke patients	PDF activity generator on the web, and PC with pre-installed rehabilitation and software (Reh@City v2.0). Duration: 12 sessions	The results showed that VR training stimulated general cognitive functioning
	Agostini et al. (13)	Exploratory study	5 poststroke patients	Target pictures were presented once for naming on a computer screen in a random	Agostini et al. demonstrated the feasibility and efficacy of TR for retrieval in lexical deficits in patients with chronic stroke, observing results similar to face-to-face therapy
Traumatic brain Injury and TR	Cruse et al. (14)	Feasibility study	20 individuals with TBI and 20 matched healthy controls	Narrative and procedural discourse	The results supported the feasibility of TR to boost discursive skills in TBI
	Kornblith et al. (15)	Feasibility study	8 elderly TBI patients	Goal-oriented attentional self-regulation (GOALS) at-home intervention 10-session intervention	The results highlighted that the TR intervention is highly feasible and acceptable for the few patients enrolled
	Tsaousides et al. (16)	Feasibility study	7 individuals with TBI	Videoconference intervention	The authors observed high participation in the sessions, adequate acquisition of skills, and ease of use of the technology
	Raso et al. (17)	Feasibility study	22 TBI patients	Home video conferencing telehealth system	The results highlighted the potential of an advanced home video conferencing telehealth system to improve the cognitive functioning of TBI patients.
Mild cognitive impairment, dementia, and TR	Jelcic et al. (18)	Pilot RCT study	27 AD patients	Lexical-semantic stimulation via TR and unstructured cognitive stimulation treatment	Results highlighted an improvement in global cognitive functioning (i.e., MMSE score) after both lexical-semantic stimulations and in language and attentional abilities after TR training
	Vermeij et al. (19)	Pilot study	23 healthy older adults and 18 MCI patients	Online 5 weeks adaptive working memory training, and after a 3 months follow-up period	The study showed the effects of working memory TR (25 sessions over 5 weeks) in patients with amnestic MCI
	Manenti et al. (20)	Exploratory study	49 subjects with MCI	Face-to-face cognitive virtual reality rehabilitation system (VRRS) followed by cognitive telerehabilitation or at-home unstructured cognitive stimulation	The authors demonstrated the feasibility and benefits of cognitive rehabilitation provided by telerehabilitation systems
	Rossetto et al. (21)	RCT	30 subjects with mild to moderate stages of AD	ABILITY (multidomain cognitive activities for 6 weeks)	Results showed that ABILITY treatment efficiently ensures patients’ compliance with the treatment, an adaptable level of difficulty (activities tailored to patients), and a usable and safe (no adverse events) experience
	Bernini et al. (22)	Pilot study	40 individuals with subjective cognitive decline or MCI	HomeCoRe 18-remote at-home TR program	Results suggest that TR is useful for improving many cognitive abilities (logical-executive functions, attention/processing speed, working memory, and episodic memory), and does not require special technological expertise
Multiple sclerosis and TR	Messinis et al. (23)	RCT	36 individuals with MS	RehaCom (45 min session per 8 weeks period, three sessions per week, at home setting) and non-specific computer-based activities at home	These data indicated the effectiveness of computerized restorative cognitive rehabilitation applied at home in progressive MS
	Chiaravallotti et al. (24)	RCT	30 individuals with MS	Modified Story Memory Technique twice per week for 5 weeks and non-training-oriented tasks	These results showed the effectiveness of TR in specifically strengthening new learning, which can significantly improve memory performance
	Naeeni Davarani et al. (25)	RCT	60 MS patients	RehaCom for 5 weeks (two 60 min sessions per week)	The results showed that RehaCom treatment improved all studied cognitive functions at the post-test stage. This effect also remained at the follow-up stage for some cognitive functions
	Arian Darestani et al. (26)	Exploratory Study	60 patients with MS	RehaCom for 5 weeks (two 60 min sessions per week)	The authors noticed that treatment with RehaCom cognitive rehabilitation software can improve verbal fluency, and verbal learning and memory in patients with MS
	Pagliari et al. (27)	Multicentre study and RCT	70 participants with MS	30 sessions of home-based virtual reality rehabilitation system training, five sessions for a week each lasting 45 min and usual care group (30 sessions of conventional treatment, five sessions for week)	This study showed that individuals with multiple sclerosis can experience positive outcomes in terms of their quality of life and motor symptoms through telerehabilitation treatments in the physical domain
Parkinson’s disease and TR	Cornejo Thumm et al. (28)	Case reports	2 patients with PD	Telerehabilitation training program using a treadmill-virtual reality system	The authors found that patients had high training adherence, allowing for individualized motor and cognitive training
	Bianchini et al. (29)	Exploratory study	23 PD patients	5 weeks telerehabilitation program	The results showed that TR is safe, feasible, and effective on symptoms in patients with PD
	Isernia et al. (30)	Exploratory study	31 PD patients	The virtual reality human Empowerment Aging and Disability (HEAD) program 12 45 min sessions, 3 sessions/week. In clinic and at-home setting	The authors observed that TR can allow an intervention tailored to the patient’s abilities, allowing motor improvement, and non-motor skills and in the quality of life of the patient, with the maintenance of the outcomes over time
	van de Weijer et al. (31)	Pilot study	3 patients with PD	Gamified cognitive training	The authors showed that gamification can be added to traditional therapies, having positive effects in terms of personalization and adherence
	Santini et al. (32)	Exploratory study	18 elderly people with PD	Online cognitive training based on cognitive stimulation therapy	This treatment was effective on the participants’ cognitive functions, but not on their mood. The online intervention was well accepted by users

AD, Alzheimer disease; MCI, mild cognitive impairment; MS, multiple sclerosis; PD, Parkinson disease; RCT, randomized controlled trial; TBI, traumatic brain injury; TR, telerehabilitation.

## 4 Devices for TR

The term telerehabilitation refers to the delivery of rehabilitation services through information and communication technologies (33). This innovative approach extends treatment directly to patients in their homes, supplementing conventional methods (34). TR encompasses various array of rehabilitative interventions, differing in pathologies, programs, duration, and technology applications such as telephone, internet, VR, and wearable devices (35). Noteworthy advantages include financial savings and reduced travel costs associated with TR (36). Unfortunately, the current literature lacks sufficient data to identify the optimal TR model or tool (37). Nevertheless, recent TR devices commonly integrate technologies to facilitate communication between patients and operators, often incorporating VR-based tasks (38). A minimum requirement for TR systems is a camera (videoconference) to enable direct visualization and monitoring of treatments. However, the integration of sensors recording patient movements is essential for TR, as movement kinematics play a crucial role in providing real-time feedback, positively impacting cognitive function during neuromotor rehabilitation (39–42). Movement data can be utilized in real-time interaction with serious games or to assess rehabilitation progress, allowing for personalized exercises (43, 44). Numerous TR systems incorporate cognitive dimensions by leveraging commercial game platforms and affordable, non-intrusive tracking devices such as Nintendo Wii controllers or Kinect (45, 46). Kinect, for instance, plays a pivotal role in TR, not only for its visual tracking and interaction capabilities but also for providing scores crucial for medical analysis (47). Its versatility extends across a spectrum of conditions, from occupational therapy to addressing challenges like Parkinson’s disease, spinal cord injury, stroke, traumatic brain injury, and cerebral palsy. In the wearable devices domain, exemplified by the BioTrak system, TR transcends a focus on motor rehabilitation. BioTrak enhances balance rehabilitation through virtual reality technology (48). The immersive nature of BioTrak’s virtual environment extends beyond physical movements, actively engaging patients in dual-task training that enriches both cognitive and motor functions.

This holistic approach reflects a growing trend in TR, where interventions extend beyond pure motor recovery to embrace the intricate interplay between cognitive and motor capabilities. Furthermore, these TR systems, by providing augmented feedback, contribute to the enhancement of cognitive domains such as attention and memory for behavioral sequences, coupled with increased execution speed. An additional cognitive facet is the crucial aspect of motor learning facilitated by these systems. Motor learning involves acquiring the skills necessary to perform a task effectively, and TR tools play a pivotal role in this process. By providing real-time feedback, these systems enable users to understand and refine their motor performance, fostering efficient execution of tasks. This dynamic interplay between cognitive domains and motor learning underscores the multifaceted cognitive benefits offered by innovative TR technologies. In Italy, the Virtual Reality Rehabilitation System (Khymeia) is prominent, delivering cognitive and speech neurological treatments remotely through advanced technological devices (49–52). The VRRS system, designed as a hub-and-spoke system, comprises the tele-cockpit (hub) and the home tablet (spoke). The therapist, through the VRRS Tele-cockpit, manages the home tablet, guiding patient training. The VRRS tablet, delivered to the patient, contains exercises to be performed using wearable sensors (Khymu and K-Wand). This non-immersive VR tool offers tailored exercises based on patients’ clinical status, encompassing trunk, upper/lower limb, and functional exercises replicating activities of daily living.

In conclusion, the evolution of TR devices hinges on their ability to intricately intertwine cognitive components. These devices must not only integrate new functionalities to facilitate exercise assignment and evaluation but also play a pivotal role in promoting autonomous exercises with immediate and nuanced cognitive feedback. While many TR devices focus on upper limb and balance recovery, they universally incorporate VR, gaming, or visual/acoustic feedback, contributing to improvements in cognitive domains such as visuospatial and executive abilities. The immersion of exercises within virtual environments should not merely target motor skills but should be designed to actively engage and challenge cognitive domains. Successful evolution further entails a commitment to cost-effectiveness, data security, and the seamless application of user-friendly technology, ensuring that the cognitive dimensions of rehabilitation are seamlessly woven into the fabric of TR advancements.

## 5 Cognitive TR and stroke

Stroke consists in the sudden onset of signs and symptoms due to focal and/or global deficits in brain function, not attributable to other causes than cerebral vasculopathy. There are two types of stroke due to different pathogenetic mechanisms: ischemic stroke is caused by the lack of blood flow to a cerebral area due to arterial occlusion, thrombus, or embolism, whereas hemorrhagic stroke is due to the rupture of cerebral veins and blood loss. A stroke is a medical emergency that requires immediate hospitalization and early intervention to avoid complications. Facing the acute (from 0–96 h) and subacute (4–10) phases, early intervention is fundamental, with timely rehabilitation and ensuring continuity of care. Stroke causes motor, sensory, and cognitive symptoms. In particular, the prevalence of post-stroke cognitive dysfunction can present between 22 and 56% within 3 months of onset and decrease to 11% and 31% 1 year after the stroke event (53–55). The main cognitive deficits are in the domains of attention (56), memory (57), praxis (58), spatial cognition, and executive functions (59). In recent years, there has been more and more interest in stroke rehabilitation through TR, especially if associated with VR, which is useful for ensuring continuity of post-hospital care and reducing healthcare costs (10). Various studies have shown the effectiveness of TR-VR.

Torrisi et al. (10) observed the efficacy of VR rehabilitation in improving the cognitive function of 40 subacute stroke patients. Patients were trained both in the hospital (experimental group with semi-immersive VR, control group with conventional rehabilitation), and after discharge, in which the experimental group underwent semi-immersive VR with tablets at home, 2 times a week. The authors observed that VR improved global cognitive functioning and semantic and phonetic fluency. Furthermore, Lawson et al. (11) used Internet videoconferencing rehabilitation to evaluate its effectiveness compared to face-to-face methods. The study involved 28 stroke patients who underwent Zoom video conferencing and 18 individuals who received face-to-face training. The authors observed that TR was a feasible and effective method, as it could increase compensatory distance memory capacity to a greater extent than face-to-face rehabilitation. These results were confirmed by a study by Faria et al. (12). The authors performed cognitive rehabilitation training in two groups: 18 patients with chronic stroke used a PDF activity generator on the web, and 14 stroke patients performed an intervention with VR, characterized by a PC with pre-installed rehabilitation software (Reh@City v2.0), a monitor and camera with augmented reality pattern tracking software. The results showed that VR training stimulated general cognitive functioning.

Another study conducted by Morse et al. (60) used a non-immersive VR telerehabilitation system based on a 40-inch monitor, a laptop, and a motion detection sensor (Kinect). The authors carried out the study on seven patients, divided into two groups: the experimental group performed the exergames, and the control group underwent computerized neuropsychological rehabilitation therapy. The results highlighted that self-managed VR could improve the psychological well-being and motivation of patients, caregivers, and therapists. However, the study has several limitations, such as a small sample size and a lack of follow-up. Furthermore, the authors emphasized that various difficulties may arise for this type of therapy, such as cost and availability of instruments, unclear instructions, and understanding of the technology.

Another field of application of TR is the aphasic syndrome and language dysfunctions, as it has been observed that almost 30% of stroke patients present with an acute phase aphasic syndrome (61–63), with long-term consequences. Maresca et al. (4) carried out a study on 30 patients using a touch-screen tablet-based semi-immersive VR system. The authors observed that TR could be a useful tool in the treatment of aphasic patients after discharge, improving the speech, mood, and psychological well-being of stroke patients. These results have been confirmed by other studies presenting good validity and reliability of TR in the treatment of acquired language disorders, particularly for fluency disorders (64–67). Agostini et al. (50) demonstrated the feasibility and efficacy of TR for retrieval in lexical deficits in patients with chronic stroke, observing results similar to face-to-face therapy. Furthermore, Latimer et al. (13) observed that TR can be effective in patients with chronic aphasia, reducing the social burden of health services. Despite these encouraging results, the studies have small sample sizes and do not follow the recommendations of the CONSORT Statement, so new RCTs are needed to validate the promising results.

To sum up, it is interesting to note, in agreement with Cogollor et al. (68), that studies with TR-VR have important limitations related to the accessibility and costs of using intelligent technologies for daily life. However, the authors emphasized that technologies for cognitive rehabilitation can be effective and allow benefits relevant to the healthcare system and the patient. The reasons for the use of TR reported in the literature are related to the high number of stroke patients who need continuous assistance, and the need to intervene in the cognitive symptoms that reduce the autonomy of individuals affected by stroke. Finally, the patients’ need for social reintegration into their living environment. TR helps address these needs through task fulfillment, monitoring, and feedback capabilities, which create an interactive and engaging environment in the patient’s home, driving improvement in daily living and cognitive enhancement.

In recent years, to overcome the accessibility and cost issues of TR, a growing interest has been given to mobile devices, such as smartphones and tablets. MHealth applications (apps) could transform healthcare delivery, fostering effective chronic disease management, such as in adults with stroke, to support activities of daily living (69). Although the Apps may be useful for managing patients at home, the literature studies on mHealth apps consist of emerging evidence, with pilot, feasibility, and case series studies. Most of the articles, even if highlighting the potential of these tools, underline the need for further studies with larger sample sizes to demonstrate the results obtained (70–72).

## 6 Telerehabilitation and cognitive-behavioral problems in TBI

Traumatic brain injury (TBI) is brain damage caused by the sudden onset of a rapid and violent external force. TBI can lead to an alteration of consciousness, and cognitive, sensory, emotional, and behavioral deficits (73, 74). In particular, various studies report that approximately 65% of patients with moderate to severe TBI may have long-term cognitive impairment (75, 76). Cognitive impairment in TBI is present in 20% to 79% of patients (77). Rehabilitative intervention plays a fundamental role in the functional recovery of patients with head trauma. In particular, recent studies highlighted the importance of information and communication technologies to increase patient recovery through home rehabilitation, leading to the integration of the patient environment (1, 14, 15, 78, 79).

Ownsworth et al. (79) evaluated the effectiveness of TR performed via smartphone or Internet. The results showed that smartphone interventions can be useful in improving mood, emotional well-being, and QoL. De Luca et al. (14), who performed a usability study on patients with severe TBI, demonstrated that the VRRS stimulates patient motivation, promoting continuity of care in the community. Indeed, the work by the same group suggests that TR is an effective tool for improving motor and cognitive outcomes and reducing behavioral alterations in patients with SABI. Furthermore, the authors highlight that TR also has a beneficial impact on the management of distress by caregivers, promoting positive aspects of care (14).

These results have been confirmed by other studies. Cruse et al. (1) evaluated the feasibility of TR to incentivize narrative and procedural discourse with 20 individuals with TBI and 20 matched healthy controls. The results supported the feasibility of TR to boost discursive skills in TBI. Kornblith et al (15) carried out goal-oriented attentional self-regulation (GOALS) at-home intervention for elderly TBI patients to improve executive function and emotional regulation. The results highlighted that the TR intervention is highly feasible and acceptable for the few patients enrolled. Furthermore, Tsaousides et al. (16) evaluated the feasibility of a videoconferencing intervention to deliver group treatments to individuals with TBI to improve emotional regulation. The authors observed high participation in the sessions, adequate acquisition of skills, and ease of use of the technology. Finally, Raso et al. (17) highlighted the potential of an advanced home video conferencing telehealth system to improve the cognitive functioning of TBI patients.

In conclusion, according to the “INCOG 2.0 Recommendations for Telerehabilitation Treatment in Traumatic Brain Injury” (80), TR allows timely access to rehabilitation for individuals with TBI. In this sense, TR should be implemented via videoconference for direct observation of the patient’s performance, non-verbal behavior, and level of effort. Despite these indications, studies relating to the TR in cognitive and motor rehabilitation are poor with small samples. Furthermore, adequate monitoring of patient motivation or treatment sessions, as required by guidelines, is not adequately implemented.

## 7 Mild cognitive impairment, dementia and TR

Dementia is currently a global health priority at the center of the global action plan (2017–2025), requiring innovative ways to support patients and their families in managing disabilities related to the disease. It is estimated that the number of people with dementia will increase from 57.4 million cases globally in 2019 to 152.8 million cases in 2050 (81). Alzheimer’s disease (AD) is the most common and severely debilitating neurodegenerative disease, with impacts on several cognitive domains including memory and language. Given the lack of pharmacological treatments, non-pharmacological interventions to prevent and treat cognitive deficits appeared to be a priority. In the AD-continuum perspective, intervening in the predementia stage, known as mild cognitive impairment (MCI) (82, 83), could allow for identifying people at risk of developing dementia and planning timely intervention (84). Several individual or group approaches are available to counteract cognitive decline from cognitive stimulation training to multi-stimulation therapies. In this context, cognitive rehabilitation, based on one-to-one sessions with a practitioner, is the most frequent personalized intervention adopted in the clinical setting. However, cognitive rehabilitation is still today a very specialized service for a limited number of patients. In this context, digital health solutions appear as a solution for scaling up cognitive rehabilitation from a limited number of patients to broader targets. In 2019, a systematic review proposed an overview of available TR cognitive training for AD-continuum, showing only a few studies (85). Jelcic et al. (18) compared the effects of lexical-semantic stimulation via TR to a similar in-person intervention and unstructured cognitive stimulation treatment in patients with AD. Results highlighted an improvement in global cognitive functioning (i.e., MMSE score) after both lexical-semantic stimulations and in language and attentional abilities after TR training. No cognitive improvement appears after unstructured cognitive stimulation treatment. Interestingly, 86% of the patients were highly satisfied by the telerehabilitation treatment. The authors concluded that it is feasible and might even be beneficial to use technology in the cognitive rehabilitation of AD. Another pilot study showed the effects of working memory TR (25 sessions over a 5 weeks period) in patients with amnestic MCI (19). The study showed an improvement in verbal and non-verbal short-term memory, and these gains were maintained at 3 months follow-up. However, only one randomized cognitive trial of cognitive TR (12 sessions via videoconferencing) was identified. The study showed encouraging results, indicating that TR is an efficacy solution to provide cognitive improvement for patients with mild dementia or MCI with comparable efficacy to face-to-face intervention. Recently, data literature supported that the most sustainable TR model to scale up rehabilitation is the asynchronous approach that overcomes the need for constant face-to-face interaction. In this approach, the TR requires a more complex technological digital system to ensure a double-loop communication between the clinic and patients’ home. There has been little research on the efficacy of an asynchronous TR model in providing rehabilitation treatment to patients in the AD continuum. Manenti et al. (20) demonstrated the feasibility and benefits of cognitive rehabilitation provided by telerehabilitation systems. They compared face-to-face cognitive virtual reality rehabilitation system (VRRS) followed by cognitive telerehabilitation or at-home unstructured cognitive stimulation and face-to-face cognitive rehabilitation programs in patients with MCI. Cognitive VRRS-TR has comparable effects to conventional rehabilitation in improving cognitive abilities in patients with neurodegenerative diseases. Application of home-based cognitive VRRS-TR seems to induce more maintenance of the obtained gains (above in memory and executive functions domains) than home-based unstructured stimulation (20). Recently, Rossetto et al. (21) have explored the effectiveness of an additional asynchronous TR model, named ABILITY, (multidomain cognitive activities for 6 weeks) to deliver rehabilitation care in a cohort of subjects with mild to moderate stages of the AD continuum. Results showed that ABILITY treatment efficiently ensures patients’ compliance with the treatment, an adaptable level of difficulty (activities tailored to patients), and a usable and safe (no adverse events) experience. The experimental group fully followed the intervention at home, while the active control group began to disengage in the fourth week. Moreover, findings pointed to a significant short- and long-term (12 months follow-up) influence of the ABILITY treatment at various cognitive domains (such as global cognitive level, language, motor perceptual, and memory). Additionally, important effects of ABILITY were also observed for caregivers, who reported decreased perception of distress related to the career’s assistance. Moreover, a recent study has shown that TR intervention is also a usable solution for patients with mild neurocognitive disorder at risk of dementia (22). Results suggest that HomeCoRe, an 18-remote at-home TR program to improve many cognitive abilities (logical-executive functions, attention/processing speed, working memory, and episodic memory), is satisfactorily usable and user-friendly and does not require special technological expertise.

Overall, the studies right now support the feasibility and pilot benefits of cognitive rehabilitation provided by TR. These findings call for increased application of TR to overcome the current limitations of in-person cognitive rehabilitation, reaching more individuals at risk of dementia. Despite the promising results suggesting a non-inferiority of TR cognitive, to date, the available evidence regarding cognitive TR remains limited and the quality of the clinical studies designs needs to be improved. Therefore, future studies need to identify guidelines for optimal treatment protocols in subjects with AD-continuum.

## 8 Cognitive TR and multiple sclerosis

Multiple sclerosis (MS) is a chronic immune-mediated disease of the central nervous system and a major cause of neurological disability in young adults (86). Due to the progressive course and long survival time, MS can lead to a high prevalence of disabilities with negative impacts on personal and social life (87). Cognitive impairment represents a common clinical feature in MS, affecting several domains such as information processing speed, attention, executive functions, and memory (88). It occurs in all multiple sclerosis phenotypes (89), and its prevalence varies across the lifespan since the early stage of the disease (88). It is well-known that cognitive impairment is present in 34 to ≥65% of patients with a great impact on the patient’s quality of life, including a reduction in participation in family and social activities, in maintaining work, and low compliance to treatments (90, 91). Therefore, prompt management of cognitive impairments is needed. Several pieces of evidence exist on structured cognitive training in patients with MS, able to reduce disability in cognitive performance (92). To date, two main approaches are used in cognitive rehabilitation: restorative and comparative which appeared useful in treating cognitive impairment in people both with relapsing-remitting and progressive course multiple sclerosis (23, 24). Interestingly, restorative and compensatory studies have shown that improved cognition is associated with changes in brain activation and connectivity (93). The restorative approach relies on repetitive training for specific cognitive functions, often via computerized tasks in clinical settings or at home via remotely guided training (94). A meta-analysis including 20 randomized controlled trials found a moderate effect size among treated patients (95). RehaCom has emerged as the computerized program most studied for people with multiple sclerosis with short- and long-term benefits in attention, cognitive processing speed, memory, and executive function (25, 26, 96–99). On the other side, strategy-based compensatory approaches emphasize manualized behavioral therapy that is administered by a therapist for individuals or groups. The modified Story Memory Technique was the first effective compensatory approach (i.e., use of context and imagery as strategies to improve the retention of information) to be published, providing class I evidence for efficacy (100). Despite the efficacy of cognitive rehabilitation, people with multiple sclerosis often have difficulty in achieving outpatient rehabilitation services because of economic, geographic, and social distancing difficulties. In this context, TR could meet the patient’s need to reconcile long-lasting programs with a social and productive life, promoting continuity of care at home via digital healthcare (101). Studies have provided evidence that telemedicine and TR in MS are beneficial, cost-effective, and satisfactory for both patients and healthcare professionals. A systematic review has investigated if patients with MS can benefit from TR approaches in the treatment of motor, cognitive, and participation symptoms (102). The authors introduced the concept of an “integrated TR approach” to refer to rehabilitative care outside of the hospital setting in which technology allowed for the double communication loop between the hospital and the patient. Through this communication, patients’ performance may be remotely monitored, and the patients can receive feedback. The authors stated that the lack of a “double loop” renders the intervention equivalent to a prescription of home exercises without a real rehabilitative component. The review showed that patients with MS benefit from TR in the treatment of motor symptoms. This result was mainly due to the motor disability outcome, while the effect on mobility and balance was moderate. On the contrary, low-quality evidence appears for the beneficial effect of the TR approach on cognition (102). The effect of TR on overall cognitive outcomes, calculated from five studies, was small and non-significant. Similar results were obtained considering targeted cognitive domains, specifically executive functions, processing speed, verbal fluency, memory, and working memory. This work has also evidenced the lack of guidelines in cognitive TR protocol that appears widely heterogeneous (e.g., training for specific cognitive functions vs. associated with interventions for compensatory/coping strategies) (102).

Recently, there has been a growing interest in combining cognitive rehabilitation with other interventions (e.g., neuromodulation techniques or aerobic exercise) to maximize effects in the TR setting. A promising recent multi-center study conducted by the Italian Network of Tele-Neurorehabilitation has shown that a dual-task virtual reality TR system could guarantee an improvement, beyond in motor domain, in cognitive abilities (e.g., memory, attention, processing speed, executive functions, and visuospatial abilities), with effects comparable to conventional intervention (27). Another study has investigated the feasibility of the TR protocol combining training exercises with tDCS at home (103). Results reported that participants rated the difficulty of clinic attendance as moderately to significantly difficult, while they had 95% treatment compliance at the TR program with 93% of participants reporting satisfaction with the at-home treatment. These combined approaches, although promising, require further research to create evidence-based guidelines for best practices. Moreover, as a recent RIMS (European network for rehabilitation in MS RIMS) survey suggests, the integration of TR health solutions into clinical practice requires more focus on education on the potential of technologies for rehabilitation and streamlining of the national healthcare system reimbursement procedures for the TR use (104).

## 9 Cognitive TR in Parkinson’s disease

Parkinson’s disease (PD) is one of the most common neurodegenerative disorders caused by the degeneration of the nigrostriatal system (104). Although the main symptoms of PD are motor, and cognitive dysfunctions are found in about 60% of patients with PD, mainly involving executive functions, attention, and visuospatial skills (105–107). In recent years, it has been shown that new technologies such as TR, especially in combination with VR, can lead to promising results in improving functional outcomes in PD (28–32, 108–111). There are few studies on the effects of TR on cognitive functions in patients with PD. Cornejo Thumm et al. (28) evaluated the effectiveness of TR with VR on two patients with PD. The authors found that patients had high training adherence, allowing for individualized motor and cognitive training. In line with these results, Bianchini et al. (29) carried out a study on the feasibility and effectiveness of TR in patients with mild to moderate PD. The authors recruited twenty-three PD patients who performed a 5 weeks telerehabilitation program. The results showed that TR is safe, feasible, and effective on symptoms in patients with PD.

Moreover, Isernia et al. (30) observed that TR can allow an intervention tailored to the patient’s abilities, allowing an improvement in motor, and non-motor skills and in the quality of life of the patient, with the maintenance of the outcomes over time.

A further interesting aspect is the spread of low-cost rehabilitation methods with the use of serious games and VR Apps that can be downloaded onto the patient’s smartphone. Regarding serious games, Mantovani et al. (109) highlighted that telerehabilitation programs can be effective options to support cognitive neurorehabilitation, allowing intensive and effective rehabilitation, even for people with reduced mobility or far from hospitals. In line with these studies, van de Weijer et al. (31) focused on gamified cognitive training in patients with PD. The authors showed in 3 patients with PD of different ages, with different stages of the disease, and of different backgrounds, that gamification can be added to traditional therapies, having positive effects in terms of personalization and adherence. In another study by the same authors on patients with PD with mild cognitive impairment, it was shown that home-gamified cognitive rehabilitation shows acceptable feasibility in patients with PD (110). Regarding smartphone apps, Lee et al. (111) observed in 29 patients with PD that, despite motor impairment, the use of smartphone technology to train the cognitive ability to resist interference can be effective. Santini et al. (32) carried out online cognitive training during COVID-19, based on cognitive stimulation therapy adapted to PD, on the cognitive domains and moods of 18 elderly people with PD. This treatment was effective on the participants’ cognitive functions, but not on their mood. Despite some initial problems with the technology, the online intervention was well accepted by users.

To sum up, TR may be effective in improving cognitive and motor outcomes in PD patients. Studies indicate that the patient demonstrates good acceptability of TR tools and high adherence to therapy. However, few studies have been conducted in this area, so more literature is needed to demonstrate the potential benefit of this type of rehabilitation.

## 10 Discussion and future perspective

To the best of our knowledge, this is the first consensus paper among multiple expert researchers that comprehensively examines TR in different neurological diseases. We aimed to evaluate the potential of this tool in neurological rehabilitation. Indeed, the results supported the efficacy and feasibility of TR with good adherence and no adverse events among patients. In fact, we found that patients with neurological disorders may benefit from TR cognitive rehabilitation, especially for those with stroke. It has been observed that TR is a feasible and effective method, as it can increase patients’ cognitive abilities and psychological well-being to equal or greater modality than in-person rehabilitation (11). Several authors have highlighted that TR applied to cognitive dysfunctions in neurological pathologies can improve executive functions, attention, memory skills, and quality of life with a more rapid return to independence. In particular, the use of TR in combination with VR can maximize the cognitive results of neurological patients, enhancing the effects of the treatment and neural plasticity and facilitating a rapid and complete recovery of the patient. Moreover, we have observed that TR in the cognitive field offers various possibilities, above all it allows the therapist to provide various technological rehabilitation services with continuous monitoring of the patient at home. Indeed, some tools, such as the VRRS, are capable of providing motor (51), cognitive, and linguistic (50, 52). VR treatments via advanced remote technological devices (51). Good outcomes are also achieved after commercial gaming platforms with low-cost non-intrusive tracking devices, such as the Nintendo Wii (45), Kinect (46), or robotic devices that use TR. Interestingly, these tools were created for motor rehabilitation and, then, extended to cognitive domains. This was possible because, often using VR allows us to improve both motor and cognitive skills, encouraging the effectiveness of the intervention. Thus, TR can play a fundamental role in the rehabilitation of the neurological patient, as highlighted during the global pandemic. In fact, TR has allowed us to overcome the limits posed by social distancing and to reach even remote places, especially through the use of cheaper tools such as smartphones, web, and video calls (32). In light of these considerations, the stimulation of cognitive function during the COVID-19 health emergency has allowed therapists to exploit the potential of TR, with remote treatments to improve cognitive performance, regardless of the diagnosis, the specific cognitive profile, and participants and the age of the patient (adult or elderly) (112). This has made it possible to stimulate alternative activities that involve and motivate patients, promoting a better quality of life (113). People could stay at home, improving their cognitive performance without exposing themselves to the risk of infection (2). Indeed, in neurological patients, to implement continuity in rehabilitation care is essential to maintain and encourage functional recovery and an adequate daily life (66, 113).

Moreover, it has been shown that prolonged and repeated therapies over time can promote long-lasting and significant improvements, so allowing patients to easily access rehabilitation is a central issue for promoting motor and cognitive recovery. However, as proposed by Hao et al. (114), TR must be conceived as an umbrella that includes multiple types of interventions, with such high variance that makes it complex to compare the present studies and thus be able to draw definitive conclusions. In our consensus, we have collected evidence of the effectiveness of different tools, such as telephone, videoconferencing, video recordings, internet, and virtual reality systems (115). Then, we are not able to state if one tool is more effective than others, concluding that TR may be a valuable way of delivering cognitive rehabilitation at a distance independently of the used tool.

Another problem regarding the literature on TR is the use of very different evaluation methods. Veras et al. (116) in a review on the use of post-stroke TR identified more than 50 outcome measures in 28 studies, where the most used were Fugl–Meyer and the Box and Block Test. Also in the cognitive field, outcome measures as well as patient population are various, and this makes the studies not so easy to compare. In detail, stroke is the most studied disease using TR, and MoCA is among the most commonly used outcome measures. The other cognitive components are assessed by heterogeneous tests, such as trail making tests or attentional matrices, or using the assessments implemented by the TR device itself.

Another interesting aspect that should be better investigated is the patient’s perception and motivation. Studies rarely consider motivational aspects and the patient’s perception of care. In any case, our article highlighted that two features of TR should encourage patient motivation. In fact, during TR, the therapist provides supervision and monitoring of patients, especially via real-time video conferencing or weekly patient reports. Furthermore, another aspect is the playful one that TR can take on, especially if combined with VR, with highly customizable exercises in terms of difficulty and interests of the patient (114). Despite these aspects, the studies do not use measures capable of quantifying patient motivation and perception, so further studies would be useful to investigate adherence to training.

TR allows the patient to eliminate the barriers of distance and travel time, to carry out a reliable, comfortable, and safe therapy, which can be customized to the patient’s needs. Our results indicate that even without the physical presence of the therapist, TR can create effective and useful bonds to promote the patient’s functional results. The various national healthcare systems need a continuous reorganization of primary care with integration between the different levels of assistance and continuity of care, especially for neurological patients. TR can be a useful tool to maximize the provision of rehabilitation services immediately and continuously, without the healthcare professional and the patient (or two professionals) being in the same location (117).

We believe that TR services can contribute to continuous management suited to the patient’s needs. This tool can promote early and protected hospital discharge, encouraging patient monitoring and treatment at home.

Unfortunately, our results highlight several limitations of the studies present in the literature on this topic. In fact, the samples of these studies are small, therefore, they do not allow a generalization of the results obtained. One reason could be the difficulty of defining software suitable for different neurological populations and unique assessment methods. Moreover, we must state that geography and culture as well as level of education and age might interfere in making an expert consensus recommendation on tele-rehabilitation universal.

Then, this manuscript highlights the need to standardize the procedures, goals, and objectives of TR. This aspect is fundamental to implementing effective TR projects, avoiding waste of resources and inconclusive results.

It would also be useful to carry out new studies with large samples, standardized outcome measures, continuous monitoring, and follow-up procedures, including evaluation of costs and clinical outcomes. Thus, although we have highlighted that the quality of research should be improved and standardized, the present studies indicate that TR is a promising tool in the rehabilitation of neurological patients.

## 11 Conclusion

TR is a promising method for neurological patient cognitive rehabilitation, considering the barriers and restrictions of traditional in-person rehabilitative treatment (114). In fact, it is a feasible method and effective tool to potentiate patient’s functional outcomes and ensure continuity of care. Furthermore, despite being provided remotely, patients could fully enjoy the rehabilitation experience through the supervision of therapists, which new technologies can be comparable to face-to-face rehabilitation. TR could have the potential to become a very beneficial rehabilitation delivery tool.

## Author contributions

MM: Data curation, Methodology, Project administration, Supervision, Validation, Writing – original draft, Writing – review & editing. FBa: Writing – original draft, Writing – review & editing. FA: Writing – original draft, Writing – review & editing. FBo: Writing – original draft, Writing – review & editing. MC: Writing – original draft, Writing – review & editing. MD: Writing – original draft, Writing – review & editing. CL: Writing – original draft, Writing – review & editing. NN: Writing – original draft, Writing – review & editing. SL: Writing – original draft, Writing – review & editing. BS: Writing – original draft, Writing – review & editing. PT: Conceptualization, Methodology, Project administration, Supervision, Validation, Writing – original draft, Writing – review & editing. RC: Conceptualization, Funding acquisition, Methodology, Project administration, Supervision, Validation, Writing – original draft, Writing – review & editing.
